# Relationship between the mean of 24-h venous blood glucose and in-hospital mortality among patients with subarachnoid hemorrhage: A matched cohort study

**DOI:** 10.3389/fneur.2022.904293

**Published:** 2022-08-02

**Authors:** Jun-Hong Wang, Hua Li, Hong-Kuan Yang, Ru-Dong Chen, Jia-Sheng Yu

**Affiliations:** Department of Neurosurgery, Tongji Hospital, Tongji Medical College, Huazhong University of Science and Technology, Wuhan, China

**Keywords:** subarachnoid hemorrhage, mean blood glucose, admission blood glucose, in-hospital mortality, MIMIC-IV database

## Abstract

**Objective:**

The aim of this study was to explore the correlation between the mean of 24-h venous blood glucose (BG) and in-hospital mortality and all-cause mortality (ACM) in patients with subarachnoid hemorrhage (SAH).

**Methods:**

Detailed clinical information was acquired from the Medical Information Mart for Intensive IV (MIMIC-IV) database. The best cutoff value of mean BG was calculated using the X-tile program. Univariate and multivariate logistic regressive analyses were utilized to analyze the prognosis significance of mean BG, and survival curves were drawn using the Kaplan-Meier (K-M) approach. To improve the reliability of results and balance the impact of underlying confounders, the 1:1 propensity score matching (PSM) approach was utilized.

**Results:**

An overall of 1,230 subjects were selected herein. The optimal cutoff value of the mean BG for in-hospital mortality was 152.25. In addition, 367 pairs of score-matched subjects were acquired after PSM analysis, and nearly all variables' differences were balanced. K-M analysis showed that patients with mean BG ≥ 152.25 mg/dl had significantly higher in-hospital, 3-month, and 6-month mortalities compared with patients with mean BG < 152.25 mg/dl (*p* < 0.001). The multivariable logistic regressive analyses revealed that patients with mean BG ≥ 152.25 mg/dl had significantly increased in-hospital mortality compared with patients with mean BG < 152.25 mg/dl after the adjustment for possible confounders (OR = 1.994, 95% CI: 1.321–3.012, *p* = 0.001). Similar outcomes were discovered in the PSM cohort.

**Conclusion:**

Our data suggested that mean BG was related to ACM of patients with SAH. More studies are needed to further analyze the role of the mean of 24-h venous BG in patients with SAH.

## Introduction

Subarachnoid hemorrhage (SAH) is one of the major health issues, with a 30-day death rate between 18 and 40% as per previously published studies (1, 2). Survivors are usually unable to regain independence from serious disability or have difficulties in communications, retention, or execution. Therefore, discovering prediction factors of the short-term or long-term prognostic results is imperative.

Previous researchers have discovered risky factors related to unsatisfactory prognoses (3–6). BG contents are usually increased on admission posterior to SAH, which might indicate a stress reaction to the bleeding (7–11). High blood glucose (BG) is common in critically ill patients (CPs), and the association between admission high BG and death rate was broadly researched as well. Liu et al. revealed that admission BG > 142.00 mg/dl (7.91 mmol/L) was related to elevated risks of modified 30- and 90-day all-cause mortality (ACM) in CPs (12). Moreover, statistically, Eagles et al. discovered a remarkable reduction in unsatisfactory prognoses among SAH sufferers maintaining the maximal BG contents lower than a determined best cutoff of 9.2 mmol/L (13). Meanwhile, Okazaki et al. found that minimum BG levels on admission were remarkably related to unsatisfactory neurological results in SAH sufferers (14). However, admission BG contents can change quickly due to stress response and altered nutrition consumption. In addition, BG is often measured clinically when there is aggravation, which might cause sampling bias. The objective of this research was to evaluate whether, in SAH sufferers, the mean of 24-h venous BG levels on ICU admission could be a better prediction factor of in-hospital death than single admission BG level alone.

## Materials and methods

### Data sources

Herein, data were acquired from a vital public database called MIMIC-IV (15). This database contains the information of sufferers admitted to the Beth Israel Deaconess Medical Center (BIDMC) between 2008 and 2019. Posterior to the completion of the National Institutes of Health (NIH) training course and the Protecting Human Research Participants test, our team acquired relevant information from MIMIC-IV. One researcher J-HW obtained approval to exploit the database. Besides, our research was accepted by the Ethics Board of our institution. The findings herein were reported using the Strengthening the Reporting of Observational Studies in Epidemiology guidelines (16).

### Study population

The diagnosis of SAH was on the basis of the International Classification of Disease, Ninth Revision. Sufferers meeting the entire standards were selected for analyses: (1) first admission to ICU; (2) age >18 years; and (3) complete records of BG examination within the first 24 h of ICU admission. The exclusion criteria were as follows: (1) ICU patients with a length of stay <24 h and (2) only one BG information in the first 24 h in ICU.

### Data acquisition

The variates stated below were acquired from the aforesaid database for the first day of ICU admission: (1) demographical variates: sex, age, and race; (2) vital signs (refer to the abbreviation list at the end of our thesis): HR, SBP, DBP, RR, temperature, and SpO2; (3) coexisting diseases: myocardium infarction, congestion-related cardiac failure, peripheral vascular illness, cerebral vascular illness, persistent lung illness, mild hepatic illness, diabetic illness, and high blood pressure; and (4) lab events (refer to the abbreviation list at the end of our thesis): WBC, neutrophil count, monocyte count, INR, PT, APTT, and BG were identified in the first 24 h of ICU admission. If a variate was identified more than once in the first 24 h, the average was utilized. (5) Severity at admission was identified *via* the SOFA scoring, the SAPS II, APS III, and GCS. (6) Duration of ICU stay, duration of hospitalization, in-hospital death, 3-month death, and 6-month death was recorded.

### Endpoints

In-hospital death, duration of ICU stay, duration of hospitalization, 3-month death, and 6-month death were regarded as endpoints.

### Statistics

The continuous variates were displayed as average ± standard deviation (SD) or mid-value (interquartile range). The Student's *t*-test or Mann-Whitney *U*-test was used according to the normality of the distribution. Categorical variates were displayed as case quantity (%), and the chi-square test (or Fisher's exact approach) was used for analyses.

The best cutoff value of mean BG was calculated by receiver operating characteristic (ROC) curve analysis using the highest Youden index for predicting survival status. Patients were separated into two groups according to mean BG, namely, low glucose (<152.25 mg/dl) and high glucose (≥152.25 mg/dl). Our team established a generalized additive model (GAM) to identify the non-linear association between mean BG and in-hospital ACM in CPs with SAH. Moreover, our team visualized the association between mean BG and sufferers' survival *via* the Kaplan-Meier (K-M) analysis and utilized the log-rank test for assumption verification.

The univariate and multivariate regressive analyses were completed to relieve the interference of possible confounding factors in the in-hospital mortality. The screening of confounders was based on: (1) the factor exerted an impact (>10%) on the research variate; (2) certain factors might remarkably affect the outcome variate according to past experiences. (3) For univariate analysis, our team modified the variates, of which *p* < 0.05. In the crude model, no variate was modified. In Model I, age, sex, and ethnicity were modified. Model II was modified in terms of age, sex, race, HR, RR, SpO2, PT, and APTT. Based on Model II, our team modified the other four variates, including hypertension, SOFA, GCS, and DBP in our Model III. Based on Model III, our team modified those variates in Model IV, including SAPS II, APS III, diabetes, mild liver disease, INR, vasospasm, DCI, urinary tract infection, sepsis, pneumonia, and WFNS grade.

Given that the sufferer screening standards can hardly be fully stochastic, our team utilized the propensity score matching (PSM) approach to realize the equilibrium of the impact of selection bias and underlying confounders. PSM analyses were on the basis of the logistic regressive model, and the propensity scoring was computed as per age and gender. The pairs of patients with low glucose (<152.25 mg/dl) and high glucose (≥152.25 mg/dl)were acquired using 1:1 matching with a caliper of 0.01. Ultimately, an overall 734 sufferers were propensity score-matched, and 367 pairs of score-matched sufferers were obtained.

Subgroup analyses were completed through a logistic regressive model as per age (<65 and ≥65 years), sex, myocardium infarction, congestion cardiac failure, peripheral vascular illness, cerebral vascular illness, persistent lung illness, mild hepatic illness, diabetic illness, and high blood pressure. Every test was two-sided, and *p* < 0.05 had significance on statistics.

Every analysis was completed *via* the statistic program packages R 3.3.2 (http://www.R-project.org, The R Foundation) and Free Statistic program 1.1. A two-tailed test was completed, and *p* < 0.05 had significance in statistics.

## Results

### Baseline features of patients

**Figure 1:**
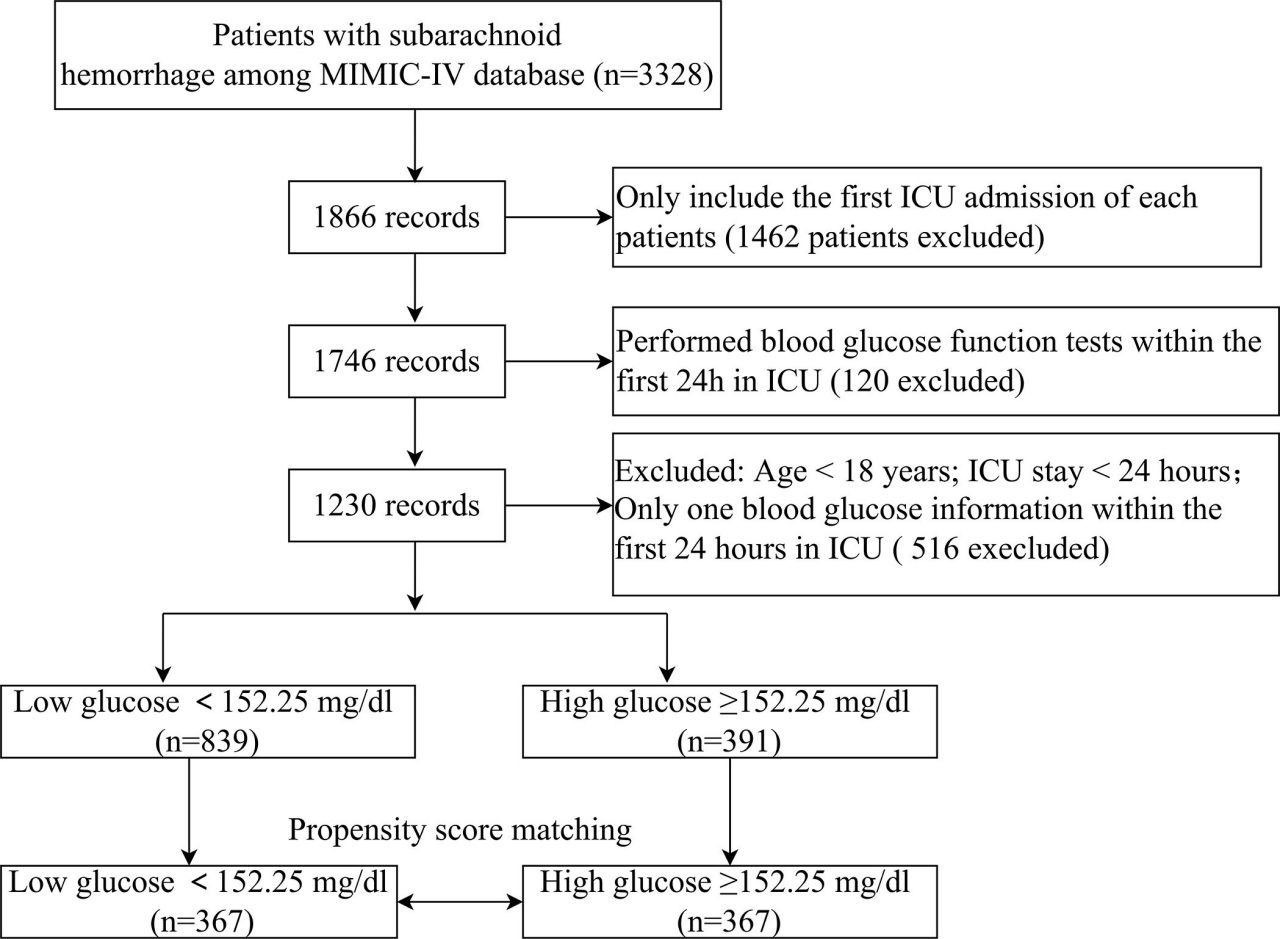
The flow chart of this study.

Overall, 1,230 sufferers meeting the standards were selected (Figure 1). The ROC curve of mean BG was plotted, and its AUC and Youden index were 0.673 (95% CI 0.630–0.715) and 0.308, respectively. The corresponding best cutoff value was 152.25, and the evaluation sensitiveness and specificness were 57.1 and 73.7%, respectively. Based on the cutoff value, 1,230 patients were divided into low glucose (mean BG < 152.25 mg/dl, *n* = 839) and high glucose (mean BG ≥ 152.25 mg/dl, *n* = 391). The demographics, vital signs, coexisting diseases, scoring, lab tests, and other related data between survivor and non-survivor groups are displayed in Supplementary Table 1. Compared with survivors, sufferers in the non-survivor group were older (age mid-value: 66.8 vs. 61.9 years, *p* < 0.001), with greater morbidity of coexisting diseases such as peripheral vascular illness and pneumonia; higher APS III scores, SAPS II scores, WFNS grade IV, and WFNS grade V; and lower GCS scores, WFNS grade I, and WFNS grade II (all *p*-values<0.05) (Supplementary Table 1).

The clinical features of CPs with SAH across two groups based on mean BG contents are displayed in Table 1. In this study, the average age of patients was 62.7 ± 17.0 years, and approximately 49.0% of them were women. Remarkable diversities were identified in age, gender, ethnicity, HR, RR, SpO2, peripheral vascular disease diabetes, mild liver disease, INR, and PT between diverse groups (*p* < 0.05). Compared with patients with mean BG <152.25 mg/dl, patients with mean BG ≥ 152.25 mg/dl were at greater risks of longer ICU stay (6.0 vs. 5.0 days, *p* < 0.001), in-hospital death (32 vs. 11.2%, *p* < 0.001), 3-month death (33.2 vs. 12%, *p* < 0.001), and 6-month death (33.8 vs. 12%, *p* < 0.001) (Table 1).

**Table 1:** The clinical characteristics in critically ill patients with SAH before and after PSM.

**Characteristic**	**Before PSM**					**After PSM**			
	**All patients**	**Low Glucose <152.25**	**High Glucose ≥152.25**	***p***		**All patients**	**Low Glucose <152.25**	**High Glucose ≥152.25**	***p***
**N**	1,230	839	391			734	367	367	
**Demographic**									
Female, *n* (%)	603 (49.0)	390 (46.5)	213 (54.5)	0.011		386 (52.6)	190 (51.8)	196 (53.4)	0.712
Age, years	62.7 ± 17.0	61.7 ± 17.8	65.0 ± 15.0	0.001		65.0 ± 14.7	65.3 ± 14.6	64.8 ± 14.9	0.696
Ethnicity, *n* (%)				0.006					0.023
Asian	42 (3.4)	24 (2.9)	18 (4.6)			26 (3.5)	11 (3)	15 (4.1)	
White	719 (58.5)	518 (61.7)	201 (51.4)			427 (58.2)	234 (63.8)	193 (52.6)	
Black	75 (6.1)	49 (5.8)	26 (6.6)			46 (6.3)	21 (5.7)	25 (6.8)	
Other	394 (32.0)	248 (29.6)	146 (37.3)			235 (32.0)	101 (27.5)	134 (36.5)	
**Vital signs**									
HR, beats/min	81.2 ± 14.5	79.6 ± 14.2	84.5 ± 14.5	< 0.001		81.9 ± 14.6	79.0 ± 13.9	84.7 ± 14.7	< 0.001
SBP, mmHg	124.1 ± 13.2	123.9 ± 13.2	124.7 ± 13.4	0.329		124.7 ± 13.2	124.8 ± 13.0	124.5 ± 13.4	0.818
DBP, mmHg	64.4 ± 9.6	64.8 ± 9.7	63.6 ± 9.2	0.048		64.3 ± 9.5	64.9 ± 9.8	63.7 ± 9.2	0.093
RR, times/min	18.0 (16.0, 20.0)	18.0 (16.0, 19.5)	19.0 (17.0, 21.0)	< 0.001		18.0 (16.0, 21.0)	18.0 (16.0, 19.0)	19.0 (17.0, 21.0)	< 0.001
Temperature, ° C	37.0 (36.8, 37.3)	37.0 (36.8, 37.3)	37.1 (36.8, 37.4)	0.357		37.0 (36.8, 37.4)	37.0 (36.8, 37.3)	37.1 (36.8, 37.4)	0.147
SpO2, %	98.0 (96.0, 99.0)	98.0 (96.0, 99.0)	98.0 (96.0, 99.0)	0.003		98.0 (96.0, 99.0)	97.0 (96.0, 99.0)	98.0 (96.0, 99.0)	< 0.001
**Comorbidities**, ***n*** **(%)**									
Myocardial infarct	145 (11.8)	93 (11.1)	52 (13.3)	0.305		100 (13.6)	52 (14.2)	48 (13.1)	0.747
Congestive heart failure	86 (7.0)	63 (7.5)	23 (5.9)	0.357		48 (6.5)	27 (7.4)	21 (5.7)	0.455
Peripheral vascular disease	811 (65.9)	531 (63.3)	280 (71.6)	0.005		509 (69.3)	246 (67)	263 (71.7)	0.2
Cerebrovascular disease	44 (3.6)	34 (4.1)	10 (2.6)	0.250		27 (3.7)	20 (5.4)	7 (1.9)	0.019
Chronic pulmonary disease	21 (1.7)	13 (1.5)	8 (2)	0.697		12 (1.6)	5 (1.4)	7 (1.9)	0.085
Mild liver disease	181 (14.7)	54 (6.4)	127 (32.5)	< 0.001		145 (19.8)	27 (7.4)	118 (32.2)	< 0.001
Diabetes	58 (4.7)	18 (2.1)	40 (10.2)	< 0.001		48 (6.5)	9 (2.5)	39 (10.6)	< 0.001
Hypertension	149 (12.1)	99 (11.8)	50 (12.8)	0.689		91 (12.4)	47 (12.8)	44 (12)	0.823
Vasospasm, *n* (%)	48 (4.8)	35 (5.3)	13 (3.8)	0.386		26 (4.2)	14 (4.7)	12 (3.8)	0.692
DCI, *n* (%)	40 (4.0)	27 (4.1)	13 (3.8)	0.983		22 (3.6)	9 (3)	13 (4.1)	0.636
Urinary tract infection, *n* (%)	85 (8.5)	63 (9.6)	22 (6.5)	0.13		47 (7.6)	25 (8.4)	22 (6.9)	0.568
Sepsis, *n* (%)	461 (46.2)	319 (48.4)	142 (42)	0.064		280 (45.5)	145 (49)	135 (42.3)	0.115
Pneumonia, *n* (%)	134 (10.9)	81 (9.7)	53 (13.6)	0.052		86 (11.7)	34 (9.3)	52 (14.2)	0.051
**Laboratory events**									
Admission glucose, mg/dL	132.0 (110.0, 161.0)	117.0 (104.0, 135.0)	183.0 (154.0, 233.0)	< 0.001		144.5 (118.0, 183.0)	119.0 (104.5, 135.0)	182.0 (154.0, 233.0)	< 0.001
WBC, 10^9^/L	199.5 (158.0, 251.8)	196.0 (158.0, 243.0)	207.0 (160.0, 264.5)	0.012		200.0 (156.2, 253.0)	193.0 (151.5, 239.0)	207.0 (160.0, 264.5)	0.004
Monocytes, 10^9^/L	26.8 (1.0, 32.7)	26.8 (0.9, 32.1)	26.8 (1.3, 32.9)	0.131		26.8 (1.1, 32.8)	26.8 (0.9, 34.8)	26.8 (1.3, 32.2)	0.090
Neutrophils, 10^9^/L	5.2 (0.1, 7.2)	5.2 (0.1, 6.7)	5.2 (0.2, 8.7)	0.021		5.2 (0.1, 7.9)	5.2 (0.1, 7.4)	5.2 (0.2, 8.6)	0.066
INR	1.1 (1.1, 1.3)	1.1 (1.1, 1.3)	1.2 (1.1, 1.4)	< 0.001		1.2 (1.1, 1.3)	1.1 (1.1, 1.2)	1.2 (1.1, 1.4)	< 0.001
PT, s	12.8 (11.8, 14.2)	12.6 (11.7, 14.0)	13.1 (12.2, 14.8)	< 0.001		12.8 (11.9, 14.4)	12.5 (11.6, 13.8)	13.2 (12.2, 14.8)	< 0.001
APTT, s	28.6 (25.9, 32.9)	28.7 (26.2, 32.9)	28.1 (25.5, 32.9)	0.165		28.5 (25.8, 32.9)	28.8 (26.3, 33.1)	28.1 (25.5, 32.9)	0.220
**Scores**									
GCS	13.0 (8.0, 14.0)	13.0 (8.0, 14.0)	12.0 (7.0, 14.0)	0.067		12.0 (7.0, 14.0)	13.0 (8.0, 14.0)	10.0 (6.0, 14.0)	< 0.001
APSIII	39.0 (28.0, 56.0)	38.0 (27.0, 55.0)	42.0 (28.0, 58.0)	0.027		44.0 (31.0, 63.0)	37.0 (27.0, 51.0)	52.0 (37.0, 74.0)	< 0.001
SAPSII	32.0 (24.0, 40.0)	31.0 (25.0, 39.0)	32.0 (23.0, 40.0)	0.910		34.0 (27.0, 43.0)	32.0 (24.0, 39.0)	37.0 (30.0, 46.0)	< 0.001
SOFA	3.0 (2.5, 3.0)	3.0 (2.2, 3.0)	3.0 (3.0, 3.0)	0.242		3.0 (2.0, 3.0)	3.0 (3.0, 3.0)	3.0 (2.0, 3.0)	0.965
WFNS Scale, *n* (%)				0.082					0.424
I	181 (14.7)	126 (15)	55 (14.1)			104 (14.2)	52 (14.2)	52 (14.2)	
II	415 (33.7)	296 (35.3)	119 (30.4)			230 (31.3)	120 (32.7)	110 (30)	
III	21 (1.7)	14 (1.7)	7 (1.8)			14 (1.9)	7 (1.9)	7 (1.9)	
IV	382 (31.1)	263 (31.3)	119 (30.4)			235 (32.0)	123 (33.5)	112 (30.5)	
V	231 (18.8)	140 (16.7)	91 (23.3)			151 (20.6)	65 (17.7)	86 (23.4)	
**Length of ICU stay, days**	5.0 (2.0, 11.0)	5.0 (2.0, 10.0)	6.0 (3.0, 13.0)	< 0.001		6.0 (2.0, 12.0)	5.0 (2.0, 10.0)	6.0 (3.0, 13.0)	0.007
**Length of hospital stay, days**	11.0 (6.0, 18.0)	10.0 (6.0, 17.0)	12.0 (5.0, 20.0)	0.335		11.0 (5.2, 18.8)	11.0 (6.0, 18.0)	12.0 (5.0, 20.0)	0.523
**In-hospital mortality**, ***n*** **(%)**	219 (17.8)	94 (11.2)	125 (32)	< 0.001		158 (21.5)	41 (11.2)	117 (31.9)	< 0.001
**3-month mortality**, ***n*** **(%)**	231 (18.8)	101 (12)	130 (33.2)	< 0.001		166 (22.6)	44 (12)	122 (33.2)	< 0.001
**6-month mortality**, ***n*** **(%)**	233 (18.9)	101 (12)	132 (33.8)	< 0.001		168 (22.9)	44 (12)	124 (33.8)	< 0.001

Values are presented as the mean ± standard deviation, median (interquartile range), or the number of patients (%).

HR, heart rate; SBP, systolic blood pressure; DBP, diastolic blood pressure; MDP, mean blood pressure; RR, respiratory rate; SpO2, percutaneous oxygen saturation; DCI, delayed cerebral ischemia; WBC, white blood cell; INR, international normalized ratio; PT, prothrombin time; APTT, activated partial thromboplastin time; GCS, Glasgow coma score; SAPS II, Simplified Acute Physiology Score II; SOFA, Sequential Organ Failure Assessment; WFNS scale, World Federation of Neurological Societies Scale; PSM, propensity score matching.

### Association between mean bg and all-cause in-hospital mortality in patients with SAH

**Figure 2:**
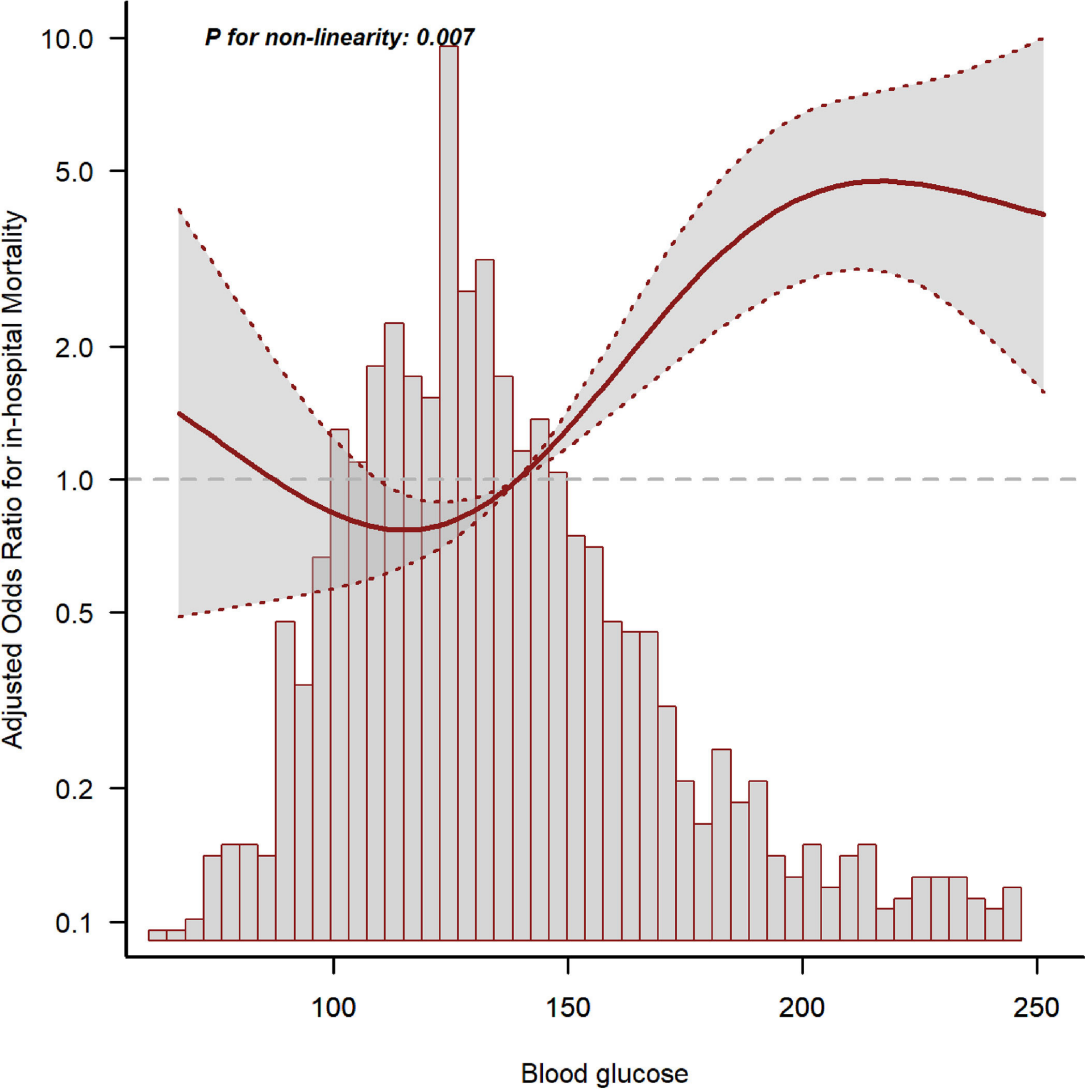
The relationship between mean blood glucose (BG) and in-hospital mortality. The solid line shows the smooth curve fit between variables. The red dotted line represents the 95% CI of the fit.

**Figure 3:**
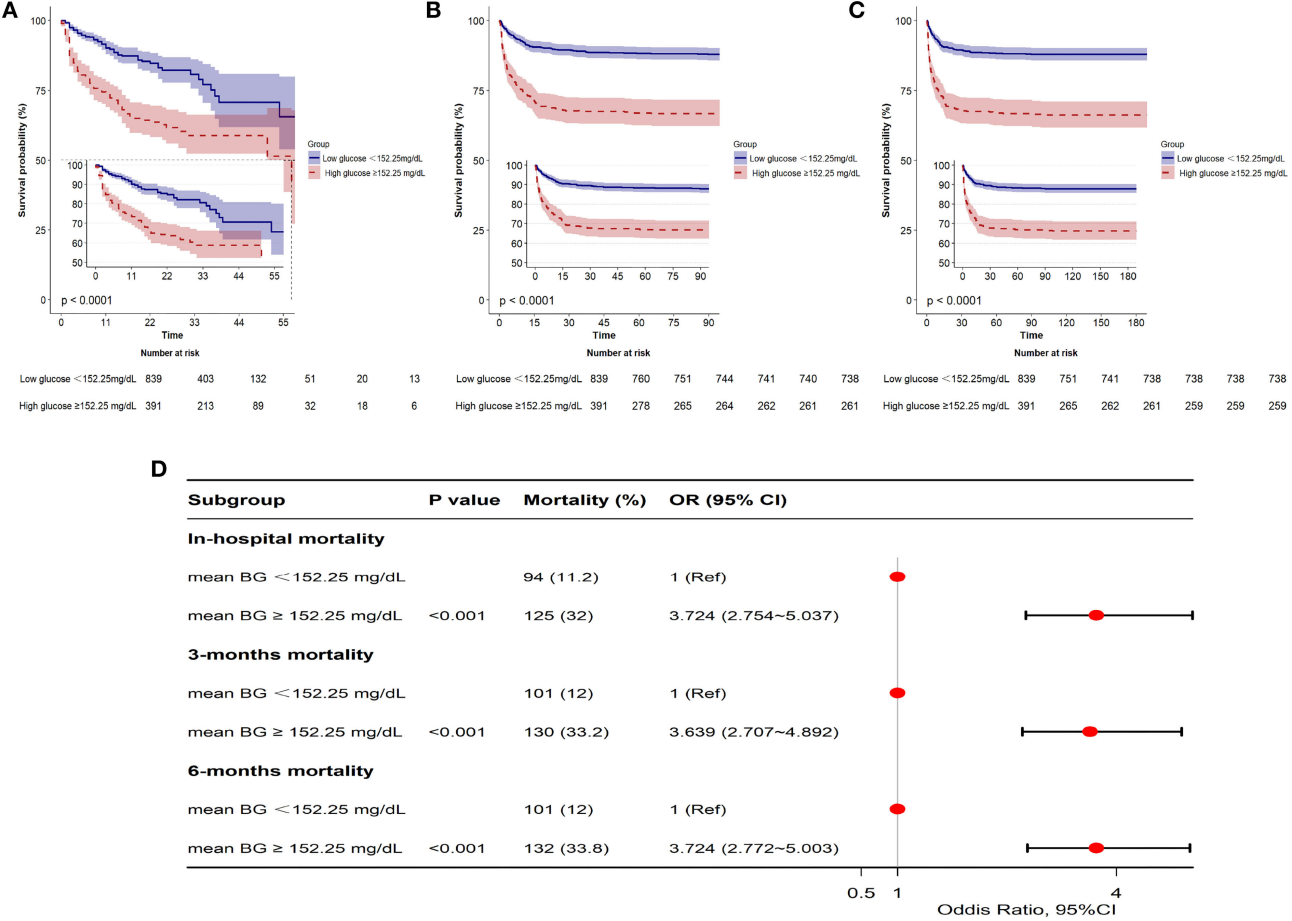
Kaplan-Meier (K-M) curves indicate the relationship between the mean BG and in-hospital mortality **(A)**, 3-month mortality **(B)**, and 6-month mortality **(C)**. Red line: high glucose ≥152.25 mg/dl; blue line: low glucose <152.25 mg/dl. Forest plot of the results based on logistic regression analysis **(D)**.

The GAM analysis revealed a U-shaped relationship between mean BG and in-hospital ACM in SAH sufferers, which revealed that aberrant mean BG might be related to elevated in-hospital death (Figure 2). The K-M curves contrasting the two groups are displayed in Figure 3. Sufferers with mean BG≥152.25 mg/dl had a significantly higher in-hospital mortality rate (Figure 3A), 3-month mortality (Figure 3B), and 6-month mortality (Figure 3C) compared with patients with mean BG < 152.25 mg/dl (*p* < 0.001). The mean BG group (≥152.25 mg/dl) exhibited a remarkably increased risk of in-hospital death (OR, 95% CI: 3.724, 2.754-5.037, *p* < 0.001), 3-month death (OR, 95% CI: 3.639, 2.707–4.892, *p* < 0.001), and 6-month death (OR, 95% CI: 3.724, 2.772–5.003, *p* < 0.001) compared with the mean BG group (< 152.25 mg/dl) (Figure 3D).

Our team utilized logistic proportion risk models to independently analyze the effects of mean BG on the risks of in-hospital death (univariable and multivariable logistic proportion risk models) (Supplementary Table 2; Table 2). In the crude model, the increase in mean BG was related to in-hospital death (OR = 1.009, 95% CI: 1.006–1.012, *p* < 0.001). In multivariate analysis, Model I was modified in terms of age, sex, and ethnicity; Model II was modified in terms of age, sex, ethnicity, HR, RR, SpO2, PT, and APTT; Model III was adjusted for Model II, DBP, hypertension, SOFA, and GCS. Based on Model III, we further modified those variates in Model IV, including SAPS II, APS III, diabetes, mild liver disease, INR, vasospasm, DCI, urinary tract infection, sepsis, pneumonia, and WFNS grade, and the results showed that patients with mean BG≥152.25 mg/dl had significantly higher in-hospital mortality compared with patients with mean BG<152.25 mg/dl (Model I: OR =3.453, 95% CI: 2.528–4.716, *p* < 0.001; Model II: OR = 2.825, 95% CI: 2.043–3.905, *p* < 0.001; Model III: 2.618, 95% CI: 1.875–3.656, *p* < 0.001; Model IV: OR = 1.994, 95% CI: 1.321–3.012, *p* = 0.001) (Table 2).

**Table 2:** Multivariate logistic regression analyses for in-hospital mortality in patients with SAH before and after PSM.

**Characteristic**	**Non-adjust model**			**Model I**			**Model II**			**Model III**		**Model IV**	
	**OR (95% CI)**	***P*-value**		**OR (95% CI)**	***P*-value**		**OR (95% CI)**	***P*-value**		**OR (95% CI)**	***P*-value**	**OR (95% CI)**	***P*-value**
**Before PSM**													
Mean blood glucose (mg/dL)	1.009 (1.006~1.012)	< 0.001		1.009 (1.006~1.012)	< 0.001		1.007 (1.004~1.010)	< 0.001		1.006 (1.003~1.009)	< 0.001	1.008 (1.004~1.012)	0.0002
Low glucose (<152.25mg/dL)	1(Ref)			1(Ref)			1(Ref)			1(Ref)		1(Ref)	
High glucose (≥152.25 mg/dL)	3.724 (2.754~5.037)	< 0.001		3.453 (2.528~4.716)	< 0.001		2.825 (2.043~3.905)	< 0.001		2.618 (1.875~3.656)	< 0.001	1.994 (1.321~3.012)	0.001
**After PSM**													
Mean blood glucose (mg/dL)	1.008 (1.005~1.011)	< 0.001		1.008 (1.005~1.011)	< 0.001		1.007 (1.004~1.01)	0.0009		1.006 (1.003~1.010)	0.0003	1.010 (1.005~1.015)	0.0001
Low glucose (<152.25mg/dL)	1(Ref)			1(Ref)			1(Ref)			1(Ref)		1(Ref)	
High glucose (≥152.25 mg/dL)	3.307 (2.264~4.83)	< 0.001		3.370 (2.302~4.933)	< 0.001		2.724 (1.833~4.049)	< 0.001		2.621 (1.742~3.945)	< 0.001	2.543 (1.485~4.357)	< 0.001

Model I, Adjusted for age, gender, and ethnicity.

Model II, Adjusted for age, gender, ethnicity, HR, RR, SpO2, PT, and APTT.

Model III, Adjusted for age, gender, ethnicity, HR, DBP, RR, SpO2, PT, APTT, hypertension, SOFA, and GCS.

Model IV, Adjusted for age, gender, ethnicity, HR, DBP, RR, SpO2, PT, APTT, hypertension, SOFA, GCS, SAPSII, APSIII, diabetes, mild liver disease, INR, vasospasm, DCI, urinary tract infection, sepsis, pneumonia, and WFNS scale.

OR, odds ratio; CI, confidence interval; PSM, propensity score matching; SAH, subarachnoid hemorrhage; HR, heart rate; RR, respiratory rate; SpO2, percutaneous oxygen saturation; PT, prothrombin time; APTT, activated partial thromboplastin time; SOFA, Sequential Organ Failure Assessment; GCS, Glasgow coma score, SAPS II, Simplified Acute Physiology Score II; APSIII, acute physiology score III; INR, international normalized ratio; DCI, delayed cerebral ischemia; WFNS scale, World Federation of Neurological Societies Scale.

### Cutoff values of mean BG and admission BG, and their correlation with in-hospital mortality

**Figure 4:**
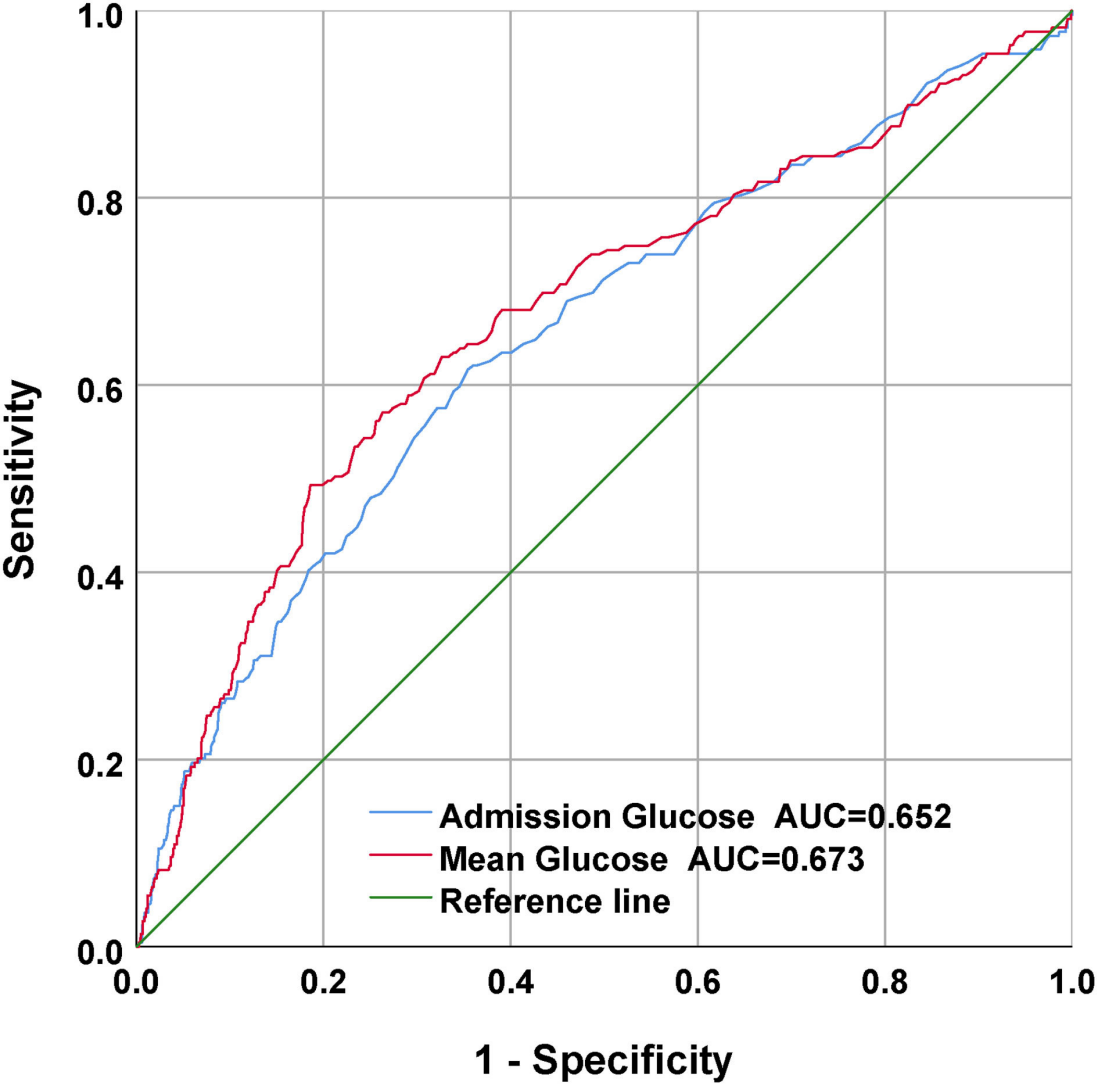
The receiver operating characteristic (ROC) curves of the predictive value of mean BG and admission BG for in-hospital mortality.

For the sake of evaluating the underlying prediction merit of the mean BG and admission BG for in-hospital mortality, ROC curve analyses were completed, and the AUC for mean BG and admission BG were 0.673 (95% CI: 0.630–0.715; *p* < 0.001) and 0.652 (95% CI: 0.610–0.694; *p* < 0.001), separately (Figure 4). The optimal cutoff values were 152.25 and 141.5, separately.

### Subgroup analysis

**Figure 5:**
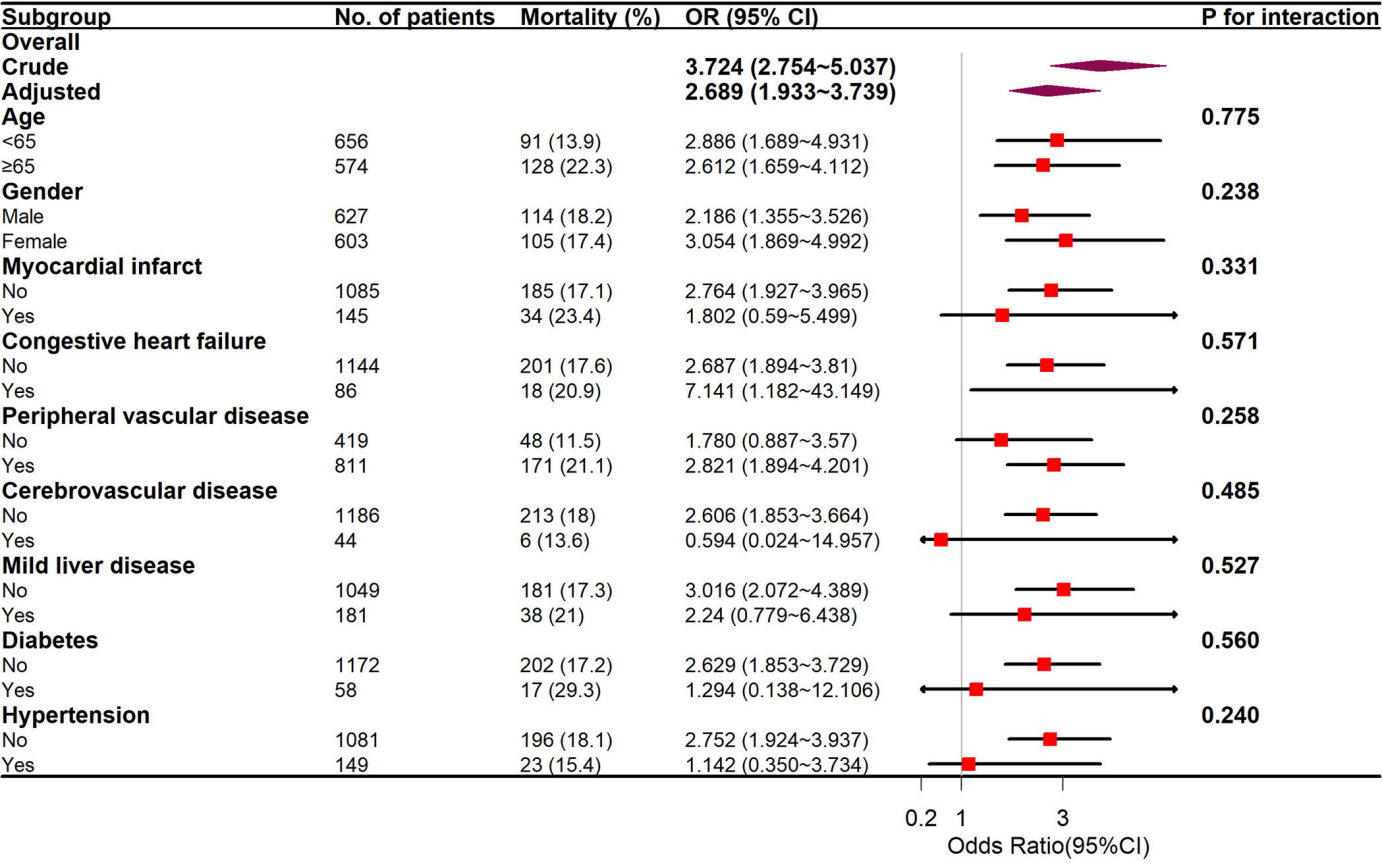
The relationship between mean BG and in-hospital mortality in subgroup analysis.

Subgroup analyses of age (<65 and ≥65 years), sex, and coexisting diseases were utilized to compare the in-hospital mortality between the two groups, and the outcomes are displayed in Figure 5. The interplay between the mean BG and the entire subgroup factors was studied and no remarkable interplay was identified. (*p* > 0.05).

### Prognosis value of mean BG after PSM

Given the imbalanced baseline features between the two groups, our team completed a 1:1 ratio PSM to realize the equilibrium of the latent confounders, and 367 pairs of score-matched sufferers were acquired. The baseline features of sufferers posterior to PSM analysis are displayed in Table 1. Posterior to PSM analysis, remarkable diversities between the two groups were still identified in the duration of ICU stay (6.0 vs. 5.0 days, *p* = 0.007), in-hospital death (31.9 vs. 11.2%, *p* < 0.001), 3-month death (33.2 vs. 12%, *p* < 0.001), and 6-month death (33.8 vs. 12%, *p* < 0.001).

The outcomes of multivariable logistic regressive analyses in sufferers posterior to PSM analysis revealed that mean BG ≥ 152.25 mg/dl was still an independent prediction factor of in-hospital death (Model I: OR = 3.370, 95% CI: 2.302–4.933, *p* < 0.001; Model II: OR = 2.724, 95% CI: 1.833–4.049, *p* < 0.001; Model III: OR = 2.621, 95% CI: 1.742–3.945, *p* < 0.001; and Model IV: OR = 2.543, 95% CI: 1.485–4.357, *p* < 0.001) (Table 2).

## Discussion

Our research was completed to analyze the relationship between mean BG and in-hospital death in CPs with SAH. The results showed that mean BG, as a continuous or categorical variate, was remarkably related to in-hospital death in multivariable logistic regressive analyses. In addition, statistically, our team discovered a remarkable elevation in unsatisfactory prognoses among sufferers maintaining mean BG contents higher than a determined best cutoff value of 152.25. Moreover, mean BG had a higher AUC value in the ROC analysis and had a better prognostic performance for in-hospital mortality than admission BG, which had been largely ignored in previous studies.

McIntyre et al. published a single-center retrospective cohort research based on 217 SAH sufferers, which showed that elevated mean BG levels were independently associated with worse outcomes. Higher mean BG could independently serve as a risk factor for the death rate and could best discriminate patients with SAH at risk of death (17). Those outcomes resembled our discoveries. Nevertheless, our research merely selected 217 sufferers and neglected multiple vital confounding factors, such as hypertension (18) and GCS score (19). In this study, we conducted the largest cohort study (*n* = 1,230), utilized an extended model strategy to modify the latent confounding factors, and discovered a steady association between mean BG and in-hospital death.

The reasons why we used average BG in this study are as follows. First, previous studies have demonstrated that high BG is common in CPs (12, 20–23). Frontera et al. retrospectively completed a cohort study of 281 SAH sufferers and found that high BG posterior to SAH was related to severe complicating diseases, more ICU stay, and elevated risk of death or serious disability (8). The study finished by Latorre et al. demonstrated that valid GLU management to sustain BG < 140 mg/dl was related to superior neurological results in SAH sufferers (9). However, few studies on the relationship between the mean of 24-h BG and the prognosis of subarachnoid hemorrhage were carried out. Second, admission BG contents can vary quickly due to stress response, altered nutrition consumption, catecholamine, cortisol, and the use of beta-blockers, or insulin. In addition, GLU is usually determined clinically when there is aggravation, which might give rise to sampling bias. The objective of our research was to reveal whether, in sufferers with SAH, the mean of 24-h venous BG levels on ICU admission can be a better prediction factor of in-hospital death than single admission BG level alone. Third, our results revealed that mean BG had a higher AUC value in the ROC analysis and had a better prognostic performance for in-hospital mortality than admission BG, which had been largely ignored in previous studies (Figure 4). Moreover, multivariate regressive analyses showed that mean BG was still related to in-hospital mortality after we adjusted potential risk factors (*p* < 0.05) (Table 2). Nevertheless, admission BG was not related to in-hospital mortality after we adjusted these factors (*p* > 0.05). Therefore, the average BG was utilized in this study.

A meta-analysis revealed that posterior to aneurysmal SAH, high BG contents on admission were common and that high BG on admission was related to unsatisfactory prognoses (24). There are several potential explanations for the relationship between high BG and unsatisfactory prognoses posterior to SAH. First, high BG on admission and high mean BG contents could derive from the stress response; therefore, they could merely denote the magnitude of the initial insult. Normally, it is suggested that the two main causal links related to stress high BG in acutely ill sufferers are reinforced liver GLU generation and elevated insulin resistance (25, 26). Second, high BG indicates aberrant GLU metabolic activity in diabetic illnesses or preexistent but undiscovered diabetic illnesses. A previous study revealed that diabetes mellitus increased the risk of poor outcomes following aneurysmal subarachnoid hemorrhage (27). Third, in this study, patients with high mean BG were older and had more comorbidities, all of which could be latent confounders eliciting elevated in-hospital ACM in SAH sufferers.

There were certain strengths in our research. The sample size herein was sufficient to determine a remarkable relationship between mean BG and in-hospital mortality in SAH sufferers. In addition, the in-depth analyses of covariable data enabled us to modify latent confounders which might influence the relationship between mean BG and mortality. Our study also explored the non-linear association between mean BG and poor outcomes, which indicated that aberrant mean BG might be related to elevated in-hospital mortality.

Despite the values of these findings, there remained certain deficiencies. First, this single-center research was finished retrospectively; therefore, multicenter prospective studies are required to substantiate our discoveries. Second, the data regarding the mean BG of certain sufferers were absent or not suitable for analysis. Therefore, these patients were excluded from this research, which might elicit selection bias, and this was one of the causes why the PSM method was utilized herein. Third, the mean BG could be influenced by a variety of factors such as diabetes, inflammation, and insulin, which might have biased the results.

## Conclusion

Our findings demonstrated that the mean of 24-h venous BG was associated with in-hospital mortality among patients with SAH. According to our results, compared with patients with mean BG < 152.25 mg/dl, patients with mean BG ≥ 152.25 mg/dl were at higher risk of prolonged ICU stay, in-hospital mortality, 3-month mortality, and 6-month mortality.

## Data availability statement

Publicly available datasets were analyzed in this study. This data can be found here: PhysioNet, https://physionet.org/, doi: 10.13026/s6n6-xd98.

## Ethics statement

The studies involving human participants were reviewed and approved by the Massachusetts Institute of Technology and Beth Israel Deaconess Medical Center. Written informed consent to participate in this study was provided by the participant's legal guardian/next of kin.

## Author contributions

J-SY and J-HW designed this study, analyzed the data, and wrote the manuscript. H-KY, HL, and R-DC reviewed, interpreted, and checked clinical data. All authors contributed to the article and approved the submitted version.

## Conflict of interest

The authors declare that the research was conducted in the absence of any commercial or financial relationships that could be construed as a potential conflict of interest.

## Publisher's note

All claims expressed in this article are solely those of the authors and do not necessarily represent those of their affiliated organizations, or those of the publisher, the editors and the reviewers. Any product that may be evaluated in this article, or claim that may be made by its manufacturer, is not guaranteed or endorsed by the publisher.

## Abbreviations

HR: heart rate
SBP: systolic blood pressure
DBP: diastolic blood pressure
MDP: mean blood pressure
RR: respiratory rate
SpO2: percutaneous oxygen saturation
DCI: delayed cerebral ischemia
WBC: white blood cell
INR: international normalized ratio
PT: prothrombin time
APTT: activated partial thromboplastin time
GCS: Glasgow coma score
SAPS II: Simplified Acute Physiology Score II
SOFA: Sequential Organ Failure Assessment
WFNS scale: World Federation of Neurological Societies Scale
PSM: propensity score matching
DCI: delayed cerebral ischemia
WFNS scale: World Federation of Neurological Societies Scale.
