# Supplementary material for: Relationship between the mean of 24-h venous blood glucose and in-hospital mortality among patients with subarachnoid hemorrhage: A matched cohort study
Source: Front Neurol. 2022 Aug 2;13:904293. doi: 10.3389/fneur.2022.904293 (PMC9379100; doi:10.3389/fneur.2022.904293)
Supplement: Supplementary file 1 [file Table_1.DOCX]

# Supplementary Table 1 | The baseline clinical characteristics of critically ill patients with SAH.

| **Variables** | **Total (n = 1230)** | **Survival (n = 1011)** | **Non-survival (n = 219)** | ***p-*value** |
| --- | --- | --- | --- | --- |
| **Demographic** |  |  |  |  |
| Female, n (%) | 603 (49.0) | 498 (49.3) | 105 (47.9) | 0.781 |
| Age, years | 62.7 ± 17.0 | 61.9 ± 17.0 | 66.8 ± 16.2 | < 0.001 |
| Ethnicity, n (%) |  |  |  | < 0.001 |
| Asian | 50 (3.3) | 40 (3.2) | 10 (3.9) |  |
| White | 905 (60.1) | 794 (63.4) | 111 (43.5) |  |
| Black | 97 (6.4) | 82 (6.5) | 15 (5.9) |  |
| Other | 455 (30.2) | 336 (26.8) | 119 (46.7) |  |
| **Vital signs** |  |  |  |  |
| HR, beats/minute | 81.2 ± 14.5 | 80.3 ± 14.2 | 85.4 ± 14.8 | < 0.001 |
| SBP, mmHg | 124.1 ± 13.2 | 124.3 ± 13.1 | 123.1 ± 13.9 | 0.208 |
| DBP, mmHg | 64.4 ± 9.6 | 64.7 ± 9.6 | 63.4 ± 9.4 | 0.084 |
| RR, times/minute | 18.0 (16.0, 20.0) | 18.0 (16.0, 20.0) | 19.0 (17.0, 22.0) | < 0.001 |
| Temperature, ^◦^ C | 37.0 (36.8, 37.3) | 37.0 (36.8, 37.3) | 37.0 (36.6, 37.5) | 0.856 |
| SpO2, % | 98.0 (96.0, 99.0) | 98.0 (96.0, 99.0) | 98.0 (97.0, 99.0) | < 0.001 |
| **Comorbidities, n (%)** |  |  |  |  |
| Myocardial infarct | 145 (11.8) | 111 (11) | 34 (15.5) | 0.076 |
| Congestive heart failure | 86 (7.0) | 68 (6.7) | 18 (8.2) | 0.409 |
| Peripheral vascular disease | 811 (65.9) | 640 (63.3) | 171 (78.1) | < 0.001 |
| Cerebrovascular disease | 44 (3.6) | 38 (3.8) | 6 (2.7) | 0.592 |
| Chronic pulmonary disease | 21 (1.7) | 18 (1.8) | 3 (1.4) | 1.000 |
| Mild liver disease | 181 (14.7) | 143 (14.1) | 38 (17.4) | 0.267 |
| Diabetes | 156 (12.7) | 122 (12.1) | 34 (15.5) | 0.200 |
| Hypertension | 149 (12.1) | 126 (12.5) | 23 (10.5) | 0.489 |
| Vasospasm, n (%) | 48 (4.8) | 37 (4.8) | 11 (5) | 1 |
| DCI, n (%) | 40 (4.0) | 32 (4.1) | 8 (3.7) | 0.911 |
| Urinary tract infection, n (%) | 85 (8.5) | 68 (8.7) | 17 (7.8) | 0.748 |
| Sepsis, n (%) | 461 (46.2) | 362 (46.5) | 99 (45.2) | 0.787 |
| Pneumonia, n (%) | 134 (10.9) | 100 (9.9) | 34 (15.5) | 0.021 |
| **Laboratory events** |  |  |  |  |
| Admission glucose, mg/dL | 132.0 (110.0, 161.0) | 128.0 (109.0, 154.5) | 152.0 (121.0, 203.5) | < 0.001 |
| Mean glucose, mg/dL | 135.0 (116.0, 161.0) | 132.0 (114.5, 154.5) | 159.5 (129.5, 194.0) | < 0.001 |
| WBC, 10^9^/L | 199.5 (158.0, 251.8) | 201.0 (162.0, 252.0) | 186.0 (137.5, 249.0) | 0.017 |
| Lymphocytes,10^9^/L | 76.6 (2.1, 76.6) | 76.6 (2.0, 76.6) | 76.6 (2.2, 81.6) | 0.466 |
| Monocytes, 10^9^/L | 26.8 (1.0, 32.7) | 26.8 (1.0, 31.5) | 26.8 (1.3, 39.9) | 0.094 |
| Neutrophils, 10^9^/L | 5.2 (0.1, 7.2) | 5.2 (0.1, 7.0) | 5.2 (0.2, 8.8) | 0.111 |
| INR | 1.1 (1.1, 1.3) | 1.1 (1.1, 1.2) | 1.2 (1.1, 1.5) | < 0.001 |
| PT, s | 12.8 (11.8, 14.2) | 12.6 (11.8, 13.9) | 13.7 (12.5, 16.1) | < 0.001 |
| APTT, s | 28.6 (25.9, 32.9) | 28.3 (25.8, 32.1) | 30.2 (26.7, 37.0) | < 0.001 |
| **Scores** |  |  |  |  |
| APSIII | 40.5 (29.0, 58.0) | 37.0 (27.0, 51.0) | 65.0 (45.5, 88.5) | < 0.001 |
| GCS | 13.0 (8.0, 14.0) | 13.0 (9.0, 14.0) | 7.0 (3.0, 15.0) | < 0.001 |
| SAPSII | 32.0 (25.0, 40.0) | 30.0 (23.0, 38.0) | 41.0 (34.0, 54.0) | < 0.001 |
| SOFA | 3.0 (2.0, 3.0) | 3.0 (2.0, 3.0) | 3.0 (2.0, 4.0) | < 0.001 |
| WFNS grade, n (%) |  |  |  | < 0.001 |
| Ⅰ | 181 (14.7) | 158 (15.6) | 23 (10.5) |  |
| Ⅱ | 415 (33.7) | 374 (37) | 41 (18.7) |  |
| Ⅲ | 21 (1.7) | 17 (1.7) | 4 (1.8) |  |
| Ⅳ | 382 (31.1) | 309 (30.6) | 73 (33.3) |  |
| Ⅴ | 231 (18.8) | 153 (15.1) | 78 (35.6) |  |
| **Length of ICU stay, days** | 8.0 ± 8.3 | 8.2 ± 8.5 | 6.8 ± 6.6 | 0.020 |
| **Length of hospital stay, days** | 13.9 ± 13.2 | 14.8 ± 13.0 | 9.4 ± 13.1 | < 0.001 |

Values are presented as the mean ± standard deviation, median (interquartile range), or the number of patients (%).

HR, heart rate; SBP, systolic blood pressure; DBP, diastolic blood pressure; MDP, mean blood pressure; RR, respiratory rate; SpO2, percutaneous oxygen saturation; DCI, delayed cerebral ischemia; WBC, white blood cell; INR, international normalized ratio; PT, prothrombin time; APTT, activated partial thromboplastin time; GCS, Glasgow coma score; SAPS II, Simplified Acute Physiology Score II; SOFA, Sequential Organ Failure Assessment; WFNS scale, World Federation of Neurological Societies Scale; PSM, , propensity score matching.
