# Supplementary material for: Relationship between the mean of 24-h venous blood glucose and in-hospital mortality among patients with subarachnoid hemorrhage: A matched cohort study
Source: Front Neurol. 2022 Aug 2;13:904293. doi: 10.3389/fneur.2022.904293 (PMC9379100; doi:10.3389/fneur.2022.904293)
Supplement: Supplementary file 2 [file Table_2.DOCX]

**Supplementary Table 2 | Univariate logistic regression analyses for in-hospital mortality in SAH patients.**

| **Variable** | OR (95% CI) | **P value** |
| --- | --- | --- |
| Age | 1.018 (1.009~1.027) | <0.001 |
| Gender | 0.949 (0.708~1.271) | 0.7246 |
| Ethnicity |  |  |
| White | 0.518 (0.24~1.118) | 0.0939 |
| Black | 0.842 (0.329~2.151) | 0.7186 |
| Other | 1.367 (0.633~2.952) | 0.4260 |
| HR | 1.024 (1.014~1.034) | <0.001 |
| SBP | 0.993 (0.982~1.004) | 0.2080 |
| DBP | 0.986 (0.971~1.002) | 0.0840 |
| RR | 1.15 (1.104~1.198) | <0.001 |
| Temperature | 1.009 (0.971~1.048) | 0.6518 |
| Spo2 | 1.104 (1.017~1.198) | 0.0175 |
| Hypertension | 0.824 (0.515~1.32) | 0.4207 |
| Myocardial infarct | 1.49 (0.984~2.258) | 0.0599 |
| Congestive heart failure | 1.242 (0.723~2.134) | 0.4329 |
| Peripheral vascular disease | 2.065 (1.463~2.915) | <0.001 |
| Cerebrovascular disease | 0.721 (0.301~1.728) | 0.4636 |
| Chronic pulmonary disease | 0.766 (0.224~2.624) | 0.6716 |
| Mild liver disease | 1.274 (0.861~1.886) | 0.2254 |
| Diabetes | 1.991 (1.109~3.575) | 0.0211 |
| Vasospasm | 1.059 (0.531~2.113) | 0.8705 |
| DCI | 0.884 (0.401~1.947) | 0.7593 |
| Urinary tract infection | 0.879 (0.505~1.529) | 0.6473 |
| Sepsis | 0.948 (0.702~1.281) | 0.7285 |
| Pneumonia | 1.674 (1.1~2.548) | 0.0162 |
| Admission glucose | 1.005 (1.003~1.008) | <0.001 |
| Mean glucose | 1.009 (1.006~1.012) | <0.001 |
| WBC | 0.998 (0.996~1) | 0.0362 |
| Monocytes | 1.007 (1.003~1.011) | <0.001 |
| Neutrophils | 1.035 (1.009~1.062) | 0.0076 |
| INR | 1.241 (1.07~1.438) | 0.0042 |
| PT | 1.019 (1.006~1.032) | 0.0046 |
| APTT | 1.012 (1.007~1.017) | <0.001 |
| APSIII | 1.046 (1.039~1.053) | <0.001 |
| GCS | 0.844 (0.814~0.875) | <0.001 |
| SAPSII | 1.082 (1.068~1.096) | <0.001 |
| SOFA | 1.342 (1.211~1.487) | <0.001 |
| WFNS grade |  |  |
| Ⅱ | 0.753 (0.437~1.297) | 0.3064 |
| Ⅲ | 1.616 (0.5~5.228) | 0.4227 |
| Ⅳ | 1.623 (0.978~2.693) | 0.0609 |
| Ⅴ | 3.502 (2.092~5.864) | <0.001 |
| Length of ICU stay | 0.975 (0.954~0.996) | 0.0197 |
| Length of hospital stay | 0.943 (0.925~0.962) | <0.001 |

HR, heart rate; SBP, systolic blood pressure; DBP, diastolic blood pressure; MDP, mean blood pressure; RR, respiratory rate; SpO2, percutaneous oxygen saturation; WBC, white blood cell; INR, international normalized ratio; PT, prothrombin time; APTT, activated partial thromboplastin time; GCS, Glasgow coma score; SAPS II, Simplified Acute Physiology Score II; SOFA, Sequential Organ Failure Assessment; DCI, delayed cerebral ischemia; WFNS grade, World Federation of Neurological Societies Grade; PSM, propensity score matching.
